# Poly[[diaqua­[μ_4_-4,4′-carbonyl­bis(benzene-1,2-dicarboxyl­ato)]bis­(dipyrido[3,2-*a*:2′,3′-*c*]phenazine)dicadmium(II)] monohydrate]

**DOI:** 10.1107/S1600536808013676

**Published:** 2008-05-14

**Authors:** Xiao-Huan Yuan, Wen-Zhi Zhang, Yan-Hui Chu

**Affiliations:** a Key Laboratory for Anti-fibrosis Biotherapy of Heilongjiang, Mudanjiang Medical University, Mudanjiang 157011, Heilongjiang Province, People’s Republic of China; b College of Chemistry and Chemical Engineering, Qiqihar University, Qiqihar 161006, Heilongjiang Province, People’s Republic of China

## Abstract

In the title compound, {[Cd_2_(C_17_H_6_O_9_)(C_18_H_10_N_4_)_2_(H_2_O)_2_]·H_2_O}_*n*_, the Cd^II^ atom is seven-coordinated by five O atoms from two different 4,4′-carbonyl­bis(benzene-1,2-dicarboxyl­ate) (BPTC) anions and one water mol­ecule, and by two N atoms from one chelating dipyrido[3,2-*a*:2′,3′-*c*]phenazine (*L*) ligand in a distorted penta­gonal-bipyramidal geometry. The BPTC anions link the Cd^II^ atoms, forming a one-dimensional chain structure. The *L* ligands are attached on both sides of the chain. A twofold rotation axis passes through the complex molecule. The crystal structure involves O—H⋯O hydrogen bonds.

## Related literature

For related literature, see: Li *et al.* (2007 ▶); Wu *et al.* (1997 ▶).
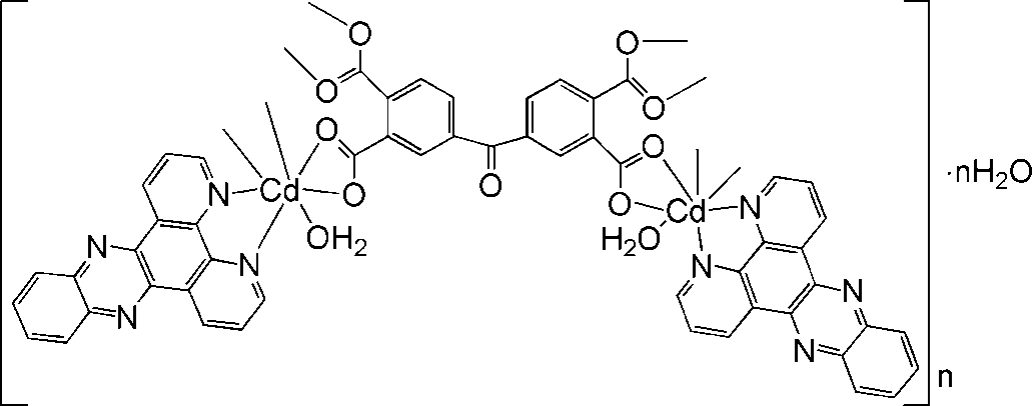


## Experimental

### 

#### Crystal data

[Cd_2_(C_17_H_6_O_9_)(C_18_H_10_N_4_)_2_(H_2_O)_2_]·H_2_O
*M* *_r_* = 1197.67
Monoclinic, 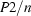

*a* = 15.698 (3) Å
*b* = 6.7028 (13) Å
*c* = 21.428 (4) Å
β = 102.45 (3)°
*V* = 2201.7 (8) Å^3^
*Z* = 2
Mo *K*α radiation
μ = 1.05 mm^−1^
*T* = 293 (2) K
0.27 × 0.24 × 0.21 mm

#### Data collection

Rigaku R-AXIS RAPID diffractometer
Absorption correction: multi-scan (*ABSCOR*; Higashi, 1995 ▶) *T*_min_ = 0.742, *T*_max_ = 0.801
20222 measured reflections
5022 independent reflections
3508 reflections with *I* > 2σ(*I*)
*R*_int_ = 0.093

#### Refinement

*R*[*F* ^2^ > 2σ(*F* ^2^)] = 0.049
*wR*(*F* ^2^) = 0.125
*S* = 1.04
5022 reflections
352 parameters
6 restraints
H atoms treated by a mixture of independent and constrained refinement
Δρ_max_ = 0.70 e Å^−3^
Δρ_min_ = −0.84 e Å^−3^

### 

Data collection: *PROCESS-AUTO* (Rigaku, 1998 ▶); cell refinement: *PROCESS-AUTO*; data reduction: *PROCESS-AUTO*; program(s) used to solve structure: *SHELXS97* (Sheldrick, 2008 ▶); program(s) used to refine structure: *SHELXL97* (Sheldrick, 2008 ▶); molecular graphics: *SHELXTL-Plus* (Sheldrick, 2008 ▶); software used to prepare material for publication: *SHELXL97*.

## Supplementary Material

Crystal structure: contains datablocks global, I. DOI: 10.1107/S1600536808013676/rz2212sup1.cif

Structure factors: contains datablocks I. DOI: 10.1107/S1600536808013676/rz2212Isup2.hkl

Additional supplementary materials:  crystallographic information; 3D view; checkCIF report

## Figures and Tables

**Table:** 

Cd1—N1	2.352 (4)
Cd1—N2	2.367 (4)
Cd1—O1	2.381 (4)
Cd1—O2	2.411 (3)
Cd1—O1*W*	2.323 (4)
Cd1—O3^i^	2.321 (4)
Cd1—O5^i^	2.572 (4)

**Table:** 

O3^i^—Cd1—O1*W*	102.56 (15)
O3^i^—Cd1—N1	84.39 (13)
O1*W*—Cd1—N1	102.54 (15)
O3^i^—Cd1—N2	154.84 (14)
O1*W*—Cd1—N2	82.23 (16)
N1—Cd1—N2	70.49 (14)
O3^i^—Cd1—O1	88.62 (13)
O1*W*—Cd1—O1	153.69 (14)
N1—Cd1—O1	102.20 (14)
N2—Cd1—O1	97.78 (14)
O3^i^—Cd1—O2	119.58 (12)
O1*W*—Cd1—O2	99.59 (13)
N1—Cd1—O2	142.59 (13)
N2—Cd1—O2	83.19 (13)
O1—Cd1—O2	54.58 (12)
O3^i^—Cd1—O5^i^	53.13 (13)
O1*W*—Cd1—O5^i^	82.76 (15)
N1—Cd1—O5^i^	136.94 (13)
N2—Cd1—O5^i^	151.32 (14)
O1—Cd1—O5^i^	85.29 (13)
O2—Cd1—O5^i^	75.40 (12)

Symmetry code: (i) 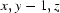.

**Table 2:** Hydrogen-bond geometry (Å, °)

*D*—H⋯*A*	*D*—H	H⋯*A*	*D*⋯*A*	*D*—H⋯*A*
O1*W*—H*W*11⋯O2^ii^	0.84 (4)	1.92 (3)	2.731 (5)	163 (6)
O1*W*—H*W*12⋯O5^ii^	0.84 (4)	2.23 (4)	2.892 (6)	136 (5)
O2*W*—H*W*22⋯O1^i^	0.85 (2)	2.14 (8)	2.913 (5)	152 (15)

Symmetry codes: (i) 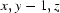; (ii) 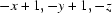.

**Fig. 1.**
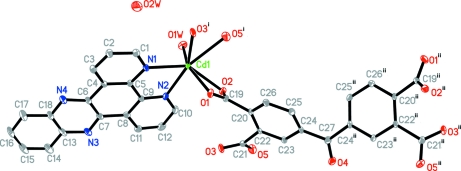
The structure of the title compound , showing the atom-numbering scheme. Displacement ellipsoids are drawn at the 30% probability level. Symmetry codes: (i) x, y - 1, z; (ii) 0.5 - x, y, 0.5 - z.

**Fig. 2.**
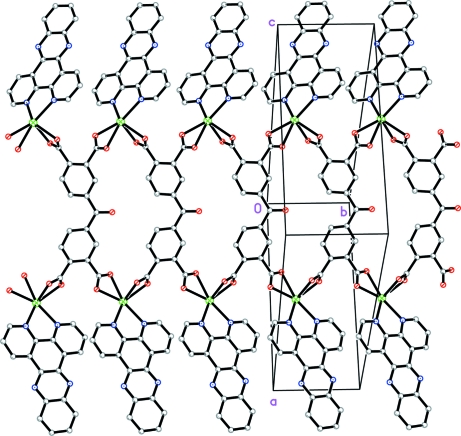
View of the chain structure of the title compound.

## supplementary crystallographic information

### Comment

Dipyrido[3,2-a:2',3'-c]phenazine (*L*) has been widely used to recognize the secondary structure of DNA in rutenium(II) complexes (Wu *et al.*, 1997). Recently, the *L* ligand has received intense interest in the chemistry of coordination polymers (Li *et al.*, 2007). In the present paper, we selected H_4_BPTC = 3,3',4,4'-benzophenone tetracarboxylic acid as a bridging ligand and *L* as a chelating ligand, generating a new cadmium(II) coordination polymer, [Cd_2_(*L*)_2_(BPTC)(H_2_O)_2_]^.^2H_2_O.

Selected bond lengths and angles for the title compound are given in Table 1. Each Cd^II^ atom is seven-coordinated by five O atoms from two different BPTC anions and one water molecule, and two N atoms from one chelating *L* ligand in a distorted pentagonal bipyramidal coordination geometry (Fig. 1). The BPTC anions link the Cd^II^ atoms to form a one-dimensional chain structure (Fig. 2). The *L* ligands are attached on both sides of the chain. Intermolecular O—H···O H-bonds (Table 2) and the π–π interactions (between *L* ligands of neighboring chains, with the shortest atom-to-atom distance of 3.43 (2) Å) stabilize the crystal structure.

### Experimental

Dipyrido[3,2 - a:2',3'-c]-phenazine (0.5 mmol) and 3,3',4,4'-benzophenone tetracarboxylic acid (0.25 mmol) were mixed with an aqueous solution (12 ml) of cadmium chloride dihydrate (0.5 mmol) with stirring. The solution was heated in a 25 ml Teflon-lined reaction vessel at 390 K for 120 h and then cooled to room temperature over a period of 16 h. Colourless crystals of the title compound were collected.

### Refinement

All H atoms on C atoms were positioned geometrically (C—H = 0.93 Å) and refined as riding, with *U*_iso_(H) = 1.2*U*_eq_(C). The water H atoms were located in a difference Fourier map and refined with a distance restraint of O–H = 0.85 Å, and with *U*_iso_(H) = 1.2*U*_eq_(O).

### Crystal data

**Table:**

[Cd_2_(C_17_H_6_O_9_)(C_18_H_10_N_4_)_2_(H_2_O_1_)_2_]·H_2_O	*F*_000_ = 1196
*M**_r_* = 1197.67	*D*_x_ = 1.807 Mg m^−^^3^
Monoclinic, *P*2/*n*	Mo *K*α radiation λ = 0.71073 Å
Hall symbol: -P 2yac	Cell parameters from 13844 reflections
*a* = 15.698 (3) Å	θ = 3.0–27.5º
*b* = 6.7028 (13) Å	µ = 1.05 mm^−^^1^
*c* = 21.428 (4) Å	*T* = 293 (2) K
β = 102.45 (3)º	Block, colourless
*V* = 2201.7 (8) Å^3^	0.27 × 0.24 × 0.21 mm
*Z* = 2	

### Data collection

**Table:**

Rigaku R-AXIS RAPID diffractometer	5022 independent reflections
Radiation source: rotating anode	3508 reflections with *I* > 2σ(*I*)
Monochromator: graphite	*R*_int_ = 0.093
Detector resolution: 10.0 pixels mm^-1^	θ_max_ = 27.5º
*T* = 293(2) K	θ_min_ = 3.0º
ω scans	*h* = −20→18
Absorption correction: multi-scan(ABSCOR; Higashi, 1995)	*k* = −8→8
*T*_min_ = 0.742, *T*_max_ = 0.801	*l* = −27→27
20222 measured reflections	

### Refinement

**Table:**

Refinement on *F*^2^	Secondary atom site location: difference Fourier map
Least-squares matrix: full	Hydrogen site location: inferred from neighbouring sites
*R*[*F*^2^ > 2σ(*F*^2^)] = 0.049	H atoms treated by a mixture of independent and constrained refinement
*wR*(*F*^2^) = 0.125	*w* = 1/[σ^2^(*F*_o_^2^) + (0.0614*P*)^2^] where *P* = (*F*_o_^2^ + 2*F*_c_^2^)/3
*S* = 1.04	(Δ/σ)_max_ < 0.001
5022 reflections	Δρ_max_ = 0.70 e Å^−^^3^
352 parameters	Δρ_min_ = −0.84 e Å^−^^3^
6 restraints	Extinction correction: none
Primary atom site location: structure-invariant direct methods	

### Special details

**Table:**

Geometry. All e.s.d.'s (except the e.s.d. in the dihedral angle between two l.s. planes) are estimated using the full covariance matrix. The cell e.s.d.'s are taken into account individually in the estimation of e.s.d.'s in distances, angles and torsion angles; correlations between e.s.d.'s in cell parameters are only used when they are defined by crystal symmetry. An approximate (isotropic) treatment of cell e.s.d.'s is used for estimating e.s.d.'s involving l.s. planes.
Refinement. Refinement of *F*^2^ against ALL reflections. The weighted *R*-factor *wR* and goodness of fit *S* are based on *F*^2^, conventional *R*-factors *R* are based on *F*, with *F* set to zero for negative *F*^2^. The threshold expression of *F*^2^ > σ(*F*^2^) is used only for calculating *R*-factors(gt) *etc*. and is not relevant to the choice of reflections for refinement. *R*-factors based on *F*^2^ are statistically about twice as large as those based on *F*, and *R*- factors based on ALL data will be even larger.

### Fractional atomic coordinates and isotropic or equivalent isotropic displacement parameters (Å^2^)

**Table:**

	*x*	*y*	*z*	*U*_iso_*/*U*_eq_	Occ. (<1)
C1	0.7768 (4)	0.0149 (8)	0.1740 (2)	0.0342 (12)	
H1	0.7343	−0.0638	0.1861	0.041*	
C2	0.8642 (3)	−0.0462 (8)	0.1917 (3)	0.0377 (13)	
H2	0.8794	−0.1597	0.2166	0.045*	
C3	0.9266 (4)	0.0645 (8)	0.1717 (3)	0.0367 (12)	
H3	0.9848	0.0255	0.1819	0.044*	
C4	0.9015 (3)	0.2375 (7)	0.1356 (2)	0.0286 (11)	
C5	0.8145 (3)	0.2937 (7)	0.1223 (2)	0.0261 (10)	
C6	0.9661 (3)	0.3643 (8)	0.1135 (2)	0.0319 (11)	
C7	0.9401 (4)	0.5492 (8)	0.0840 (2)	0.0339 (12)	
C8	0.8467 (4)	0.6054 (7)	0.0703 (2)	0.0316 (12)	
C9	0.7859 (3)	0.4772 (7)	0.0878 (2)	0.0281 (11)	
C10	0.6728 (4)	0.6892 (8)	0.0431 (3)	0.0423 (14)	
H10	0.6134	0.7174	0.0328	0.051*	
C11	0.8165 (4)	0.7825 (8)	0.0393 (3)	0.0398 (13)	
H11	0.8559	0.8716	0.0279	0.048*	
C12	0.7312 (4)	0.8258 (8)	0.0259 (3)	0.0502 (17)	
H12	0.7112	0.9446	0.0055	0.060*	
C13	1.0803 (4)	0.6155 (10)	0.0777 (3)	0.0455 (15)	
C14	1.1439 (5)	0.7468 (10)	0.0625 (3)	0.0604 (19)	
H14	1.1280	0.8737	0.0466	0.072*	
C15	1.2290 (5)	0.6847 (13)	0.0715 (3)	0.068 (2)	
H15	1.2708	0.7707	0.0618	0.082*	
C16	1.2537 (4)	0.4954 (12)	0.0950 (3)	0.0566 (19)	
H16	1.3116	0.4562	0.0998	0.068*	
C17	1.1943 (4)	0.3640 (12)	0.1113 (3)	0.0550 (17)	
H17	1.2119	0.2383	0.1275	0.066*	
C18	1.1052 (4)	0.4256 (9)	0.1028 (3)	0.0395 (13)	
C19	0.5216 (3)	0.5601 (7)	0.1603 (3)	0.0300 (11)	
C20	0.4599 (3)	0.6828 (7)	0.1904 (2)	0.0250 (10)	
C21	0.4935 (3)	1.0024 (7)	0.1355 (2)	0.0298 (11)	
C22	0.4394 (3)	0.8792 (7)	0.1726 (2)	0.0228 (10)	
C23	0.3716 (3)	0.9765 (7)	0.1933 (2)	0.0264 (10)	
H23	0.3585	1.1084	0.1817	0.032*	
C24	0.3230 (3)	0.8755 (7)	0.2318 (2)	0.0270 (11)	
C25	0.3484 (3)	0.6844 (7)	0.2525 (2)	0.0320 (12)	
H25	0.3201	0.6192	0.2805	0.038*	
C26	0.4164 (3)	0.5886 (7)	0.2315 (2)	0.0311 (11)	
H26	0.4324	0.4598	0.2454	0.037*	
C27	0.2500	0.9870 (11)	0.2500	0.0338 (17)	
N1	0.7518 (3)	0.1804 (6)	0.1405 (2)	0.0292 (9)	
N2	0.6995 (3)	0.5206 (6)	0.0737 (2)	0.0333 (10)	
N3	0.9946 (3)	0.6766 (7)	0.0669 (2)	0.0419 (11)	
N4	1.0483 (3)	0.2994 (7)	0.1226 (2)	0.0385 (11)	
O1	0.5763 (2)	0.4504 (5)	0.19432 (18)	0.0386 (9)	
O2	0.5089 (2)	0.5605 (5)	0.09996 (17)	0.0338 (8)	
O1W	0.5801 (3)	0.2021 (6)	−0.00419 (19)	0.0457 (10)	
HW11	0.563 (4)	0.287 (6)	−0.033 (2)	0.055*	
HW12	0.553 (3)	0.096 (5)	−0.016 (3)	0.055*	
O3	0.5736 (2)	0.9818 (6)	0.1491 (2)	0.0443 (10)	
O4	0.2500	1.1681 (8)	0.2500	0.0448 (15)	
O2W	0.7500	−0.3982 (12)	0.2500	0.082 (2)	
HW22	0.708 (7)	−0.479 (19)	0.240 (8)	0.099*	0.50
HW21	0.764 (14)	−0.40 (2)	0.2906 (11)	0.099*	0.50
O5	0.4540 (3)	1.1233 (6)	0.0948 (2)	0.0506 (11)	
Cd1	0.60585 (2)	0.27952 (5)	0.103914 (18)	0.02753 (13)	

### Atomic displacement parameters (Å^2^)

**Table:**

	*U*^11^	*U*^22^	*U*^33^	*U*^12^	*U*^13^	*U*^23^
C1	0.034 (3)	0.038 (3)	0.032 (3)	−0.002 (2)	0.010 (3)	0.007 (2)
C2	0.033 (3)	0.039 (3)	0.040 (3)	0.003 (2)	0.006 (3)	0.013 (2)
C3	0.029 (3)	0.043 (3)	0.035 (3)	0.001 (2)	0.001 (3)	0.005 (2)
C4	0.028 (3)	0.031 (3)	0.028 (2)	−0.001 (2)	0.008 (2)	−0.004 (2)
C5	0.025 (2)	0.030 (2)	0.026 (2)	0.000 (2)	0.011 (2)	0.002 (2)
C6	0.024 (3)	0.043 (3)	0.030 (3)	−0.009 (2)	0.009 (2)	0.000 (2)
C7	0.035 (3)	0.037 (3)	0.032 (3)	−0.012 (2)	0.013 (3)	−0.005 (2)
C8	0.036 (3)	0.031 (3)	0.031 (3)	−0.009 (2)	0.014 (3)	0.000 (2)
C9	0.030 (3)	0.025 (2)	0.031 (3)	−0.003 (2)	0.010 (2)	0.000 (2)
C10	0.036 (3)	0.038 (3)	0.058 (4)	0.010 (2)	0.020 (3)	0.011 (3)
C11	0.046 (3)	0.030 (3)	0.046 (3)	−0.003 (2)	0.016 (3)	0.005 (2)
C12	0.063 (4)	0.028 (3)	0.065 (4)	0.008 (3)	0.026 (4)	0.018 (3)
C13	0.034 (3)	0.066 (4)	0.036 (3)	−0.023 (3)	0.008 (3)	−0.008 (3)
C14	0.051 (4)	0.073 (5)	0.061 (4)	−0.024 (4)	0.021 (4)	0.009 (3)
C15	0.041 (4)	0.110 (6)	0.056 (4)	−0.039 (4)	0.018 (4)	−0.002 (4)
C16	0.023 (3)	0.108 (6)	0.042 (4)	−0.019 (3)	0.013 (3)	−0.011 (4)
C17	0.033 (3)	0.095 (5)	0.038 (3)	−0.007 (3)	0.008 (3)	−0.001 (3)
C18	0.024 (3)	0.065 (4)	0.030 (3)	−0.010 (3)	0.008 (2)	−0.003 (3)
C19	0.027 (3)	0.027 (2)	0.040 (3)	0.002 (2)	0.015 (3)	0.002 (2)
C20	0.015 (2)	0.032 (3)	0.029 (2)	−0.0024 (18)	0.005 (2)	0.001 (2)
C21	0.033 (3)	0.028 (3)	0.031 (3)	−0.005 (2)	0.013 (2)	−0.004 (2)
C22	0.014 (2)	0.029 (2)	0.025 (2)	−0.0026 (18)	0.004 (2)	0.0002 (19)
C23	0.023 (3)	0.028 (2)	0.030 (3)	−0.0025 (19)	0.009 (2)	0.002 (2)
C24	0.020 (2)	0.033 (3)	0.031 (3)	−0.004 (2)	0.013 (2)	−0.002 (2)
C25	0.032 (3)	0.033 (3)	0.035 (3)	−0.005 (2)	0.015 (2)	0.004 (2)
C26	0.027 (3)	0.030 (2)	0.036 (3)	0.002 (2)	0.007 (2)	0.005 (2)
C27	0.036 (4)	0.038 (4)	0.030 (4)	0.000	0.015 (4)	0.000
N1	0.023 (2)	0.031 (2)	0.035 (2)	−0.0024 (17)	0.0090 (19)	0.0029 (18)
N2	0.029 (2)	0.032 (2)	0.043 (3)	0.0060 (18)	0.016 (2)	0.0032 (19)
N3	0.034 (3)	0.048 (3)	0.044 (3)	−0.016 (2)	0.010 (2)	0.000 (2)
N4	0.029 (2)	0.052 (3)	0.034 (2)	−0.007 (2)	0.007 (2)	0.000 (2)
O1	0.029 (2)	0.043 (2)	0.044 (2)	0.0123 (17)	0.0088 (19)	0.0045 (18)
O2	0.038 (2)	0.0322 (18)	0.035 (2)	0.0063 (16)	0.0155 (18)	0.0007 (15)
O1W	0.058 (3)	0.042 (2)	0.033 (2)	0.011 (2)	0.002 (2)	0.0042 (18)
O3	0.022 (2)	0.049 (2)	0.066 (3)	−0.0033 (17)	0.020 (2)	0.006 (2)
O4	0.051 (4)	0.032 (3)	0.062 (4)	0.000	0.035 (3)	0.000
O2W	0.057 (6)	0.059 (5)	0.119 (7)	0.000	−0.007 (5)	0.000
O5	0.049 (3)	0.048 (2)	0.058 (3)	−0.003 (2)	0.018 (2)	0.023 (2)
Cd1	0.02447 (19)	0.02697 (19)	0.0338 (2)	0.00214 (15)	0.01220 (15)	0.00352 (16)

### Geometric parameters (Å, °)

**Table:**

C1—N1	1.333 (6)	C17—H17	0.9300
C1—C2	1.403 (7)	C18—N4	1.363 (7)
C1—H1	0.9300	C19—O1	1.240 (6)
C2—C3	1.370 (7)	C19—O2	1.265 (6)
C2—H2	0.9300	C19—C20	1.517 (6)
C3—C4	1.401 (7)	C19—Cd1	2.727 (5)
C3—H3	0.9300	C20—C26	1.378 (6)
C4—C5	1.386 (7)	C20—C22	1.389 (6)
C4—C6	1.478 (6)	C21—O3	1.236 (6)
C5—N1	1.365 (6)	C21—O5	1.252 (6)
C5—C9	1.455 (7)	C21—C22	1.526 (6)
C6—N4	1.335 (7)	C21—Cd1^i^	2.744 (5)
C6—C7	1.411 (7)	C22—C23	1.399 (6)
C7—N3	1.316 (6)	C23—C24	1.411 (6)
C7—C8	1.480 (7)	C23—H23	0.9300
C8—C11	1.392 (7)	C24—C25	1.385 (7)
C8—C9	1.395 (6)	C24—C27	1.489 (6)
C9—N2	1.356 (6)	C25—C26	1.400 (6)
C10—N2	1.328 (6)	C25—H25	0.9300
C10—C12	1.402 (8)	C26—H26	0.9300
C10—H10	0.9300	C27—O4	1.214 (9)
C11—C12	1.339 (9)	C27—C24^ii^	1.489 (6)
C11—H11	0.9300	Cd1—N1	2.352 (4)
C12—H12	0.9300	Cd1—N2	2.367 (4)
C13—N3	1.376 (8)	Cd1—O1	2.381 (4)
C13—C18	1.405 (9)	Cd1—O2	2.411 (3)
C13—C14	1.420 (8)	Cd1—O1W	2.323 (4)
C14—C15	1.374 (10)	O1W—HW11	0.84 (4)
C14—H14	0.9300	O1W—HW12	0.84 (4)
C15—C16	1.389 (10)	O2W—HW22	0.85 (2)
C15—H15	0.9300	O2W—HW21	0.85 (2)
C16—C17	1.381 (8)	Cd1—O3^iii^	2.321 (4)
C16—H16	0.9300	Cd1—O5^iii^	2.572 (4)
C17—C18	1.432 (8)		
			
N1—C1—C2	123.1 (5)	C22—C20—C19	121.9 (4)
N1—C1—H1	118.5	O3—C21—O5	124.2 (5)
C2—C1—H1	118.5	O3—C21—C22	117.9 (5)
C3—C2—C1	118.7 (5)	O5—C21—C22	117.8 (5)
C3—C2—H2	120.6	O3—C21—Cd1^i^	57.2 (3)
C1—C2—H2	120.6	O5—C21—Cd1^i^	68.8 (3)
C2—C3—C4	119.0 (5)	C22—C21—Cd1^i^	162.6 (3)
C2—C3—H3	120.5	C20—C22—C23	120.1 (4)
C4—C3—H3	120.5	C20—C22—C21	122.3 (4)
C5—C4—C3	119.1 (4)	C23—C22—C21	117.4 (4)
C5—C4—C6	119.4 (4)	C22—C23—C24	120.4 (4)
C3—C4—C6	121.5 (5)	C22—C23—H23	119.8
N1—C5—C4	121.9 (4)	C24—C23—H23	119.8
N1—C5—C9	117.0 (4)	C25—C24—C23	118.4 (4)
C4—C5—C9	121.1 (4)	C25—C24—C27	124.4 (4)
N4—C6—C7	122.0 (4)	C23—C24—C27	117.1 (4)
N4—C6—C4	118.2 (5)	C24—C25—C26	120.5 (4)
C7—C6—C4	119.8 (4)	C24—C25—H25	119.7
N3—C7—C6	123.5 (5)	C26—C25—H25	119.7
N3—C7—C8	117.1 (5)	C20—C26—C25	120.8 (5)
C6—C7—C8	119.4 (4)	C20—C26—H26	119.6
C11—C8—C9	118.1 (5)	C25—C26—H26	119.6
C11—C8—C7	122.2 (4)	O4—C27—C24^ii^	120.1 (3)
C9—C8—C7	119.7 (5)	O4—C27—C24	120.1 (3)
N2—C9—C8	121.2 (5)	C24^ii^—C27—C24	119.7 (6)
N2—C9—C5	118.5 (4)	C1—N1—C5	118.1 (4)
C8—C9—C5	120.2 (5)	C1—N1—Cd1	124.5 (3)
N2—C10—C12	122.1 (5)	C5—N1—Cd1	117.2 (3)
N2—C10—H10	119.0	C10—N2—C9	119.1 (4)
C12—C10—H10	119.0	C10—N2—Cd1	124.5 (4)
C12—C11—C8	120.6 (5)	C9—N2—Cd1	116.3 (3)
C12—C11—H11	119.7	C7—N3—C13	115.5 (5)
C8—C11—H11	119.7	C6—N4—C18	115.4 (5)
C11—C12—C10	118.9 (5)	C19—O1—Cd1	92.2 (3)
C11—C12—H12	120.6	C19—O2—Cd1	90.2 (3)
C10—C12—H12	120.6	Cd1—O1W—HW11	123 (4)
N3—C13—C18	121.0 (5)	Cd1—O1W—HW12	117 (4)
N3—C13—C14	119.1 (6)	HW11—O1W—HW12	107 (3)
C18—C13—C14	119.9 (6)	C21—O3—Cd1^i^	96.2 (3)
C15—C14—C13	119.4 (7)	HW22—O2W—HW21	105 (3)
C15—C14—H14	120.3	C21—O5—Cd1^i^	84.2 (3)
C13—C14—H14	120.3	O3^iii^—Cd1—O1W	102.56 (15)
C14—C15—C16	121.0 (6)	O3^iii^—Cd1—N1	84.39 (13)
C14—C15—H15	119.5	O1W—Cd1—N1	102.54 (15)
C16—C15—H15	119.5	O3^iii^—Cd1—N2	154.84 (14)
C17—C16—C15	121.6 (6)	O1W—Cd1—N2	82.23 (16)
C17—C16—H16	119.2	N1—Cd1—N2	70.49 (14)
C15—C16—H16	119.2	O3^iii^—Cd1—O1	88.62 (13)
C16—C17—C18	118.6 (7)	O1W—Cd1—O1	153.69 (14)
C16—C17—H17	120.7	N1—Cd1—O1	102.20 (14)
C18—C17—H17	120.7	N2—Cd1—O1	97.78 (14)
N4—C18—C13	122.3 (5)	O3^iii^—Cd1—O2	119.58 (12)
N4—C18—C17	118.0 (6)	O1W—Cd1—O2	99.59 (13)
C13—C18—C17	119.6 (5)	N1—Cd1—O2	142.59 (13)
O1—C19—O2	122.6 (4)	N2—Cd1—O2	83.19 (13)
O1—C19—C20	119.8 (5)	O1—Cd1—O2	54.58 (12)
O2—C19—C20	117.3 (4)	O3^iii^—Cd1—O5^iii^	53.13 (13)
O1—C19—Cd1	60.7 (3)	O1W—Cd1—O5^iii^	82.76 (15)
O2—C19—Cd1	62.2 (2)	N1—Cd1—O5^iii^	136.94 (13)
C20—C19—Cd1	167.9 (3)	N2—Cd1—O5^iii^	151.32 (14)
C26—C20—C22	119.5 (4)	O1—Cd1—O5^iii^	85.29 (13)
C26—C20—C19	118.2 (4)	O2—Cd1—O5^iii^	75.40 (12)

Symmetry codes: (i) *x*, *y*+1, *z*; (ii) −*x*+1/2, *y*, −*z*+1/2; (iii) *x*, *y*−1, *z*.

### Hydrogen-bond geometry (Å, °)

**Table:**

*D*—H···*A*	*D*—H	H···*A*	*D*···*A*	*D*—H···*A*
O1W—HW11···O2^iv^	0.84 (4)	1.92 (3)	2.731 (5)	163 (6)
O1W—HW12···O5^iv^	0.84 (4)	2.23 (4)	2.892 (6)	136 (5)
O2W—HW22···O1^iii^	0.85 (2)	2.14 (8)	2.913 (5)	152 (15)

Symmetry codes: (iv) −*x*+1, −*y*+1, −*z*; (iii) *x*, *y*−1, *z*.
